# Alarm fatigue and perceived stress among critical care nurses in the intensive care units: Palestinian perspectives

**DOI:** 10.1186/s12912-024-01897-x

**Published:** 2024-04-23

**Authors:** Basma Salameh, Jihad Abdallah, Sameer A Alkubati, Mohammed ALBashtawy

**Affiliations:** 1 Faculty of Nursing, Arab American University, Jenin, Palestine; 2 Department of Animal Production, An-Najah National University,Nablus, Jenin, Palestine; 3 Department of Nursing, Faculty of Medicine and Health Sciences, Hodeida University, Hodeida, Yemen; 4 Princess Salma Faculty of Nursing, AL al-Bayt University, Mafraq, Jordan; 5 Department of Medical Surgical Nursing, College of Nursing, University of Hail, Hail, Saudi Arabia

## Abstract

**Objective:**

The frequency of alarms generated by monitors and other electro-medical devices is undeniably valuable but can simultaneously escalate the workload for healthcare professionals, potentially subjecting intensive care unit nurses to alarm fatigue. The aim of this study is to investigate alarm fatigue and stress levels among critical care nursing personnel. Additionally, the study aims to assess predictors for both alarm fatigue and perceived stress.

**Methodology:**

: A descriptive cross-sectional study recruited 187 Intensive Care Unit (ICU) nurses from hospitals located in the northern and central regions of the West Bank, Palestine. Data were gathered through online surveys due to logistic concerns using the Alarm Fatigue Scale and the Perceived Stress Scale. The research was conducted between November 2023 and January 2024.

**Results:**

The mean overall alarm fatigue score was 23.36 (SD = 5.57) out of 44. The study showed that 62.6% of the participating ICU nurses experience average to high degree of alarm fatigue, while 69.5% experience average to high levels of perceived stress. A significant positive Pearson correlation was found between stress and alarm fatigue (0.40, *P* < 0.01). Important predictors of alarm fatigue include perceived stress, nurse-to-patient ratio, gender, and years of experience, while important predictors of perceived stress include alarm fatigue, type of working shift and hospital unit.

**Conclusion:**

Alarm fatigue can compromise the timely intervention required to prevent adverse outcomes by causing delayed responses or missed critical alarm, which can have major ramifications for patient safety. Addressing stress is crucial for mitigating alarm fatigue and fostering a supportive work environment to ensure optimal patient care. Consequently, exploring strategies to alleviate the negative impacts of alarm fatigue on critical care nurses’ stress merits further investigation in future research studies.

## Background

Today’s healthcare organizations have access to a wide range of technologies and resources to assist everyday clinical practice, particularly in intensive care units, where patients are continuously monitored with specialized alarm systems to alert health care providers [1–3]. 

However, continuous monitors frequently produce excessive alarm warnings that may not always indicate significant changes in the patient’s clinical status. [4–5]

Every day, nurses in intensive care units (ICUs) deal with 350 alarms per bed, 85–99% of which are not actionable. [6–7] As a result, nurses often find themselves grappling with alarm fatigue, which affects their ability to react to alarms effectively and reduce their receptivity to these notifications [8–12]. Alarm fatigue poses a significant risk for critical care nurses, who serve as the frontline healthcare providers interacting directly with patients and monitoring them around the clock [13]. Critical care nurses are often subjected to excessively frequent and burdensome alarms, potentially impeding their ability to concentrate on tasks and responsibilities, leading to lapses in `attention and increased likelihood of errors [14]. Alarm fatigue, a concerning phenomenon, is triggered by the constant stream of alarms in the ICU [6, 14]. Alarm fatigue occurs when healthcare professionals become desensitized to the frequent occurrence of alarms, particularly those with non-actionable character [6]. This tendency may cause crucial alarms to be missed or responded to slowly, endangering the safety and wellbeing of patients, which could lead to patient mortality [10]. 

Increased levels of alarm fatigue may be associated with suboptimal nursing practices, such as altering alarm parameters outside of acceptable ranges, turning down alert volume, or even turning off alarm systems entirely. Such acts may put patient safety in danger [15, 16]. Recognizing the severity of this issue, The Joint Commission, reaffirmed alarm fatigue management as a global priority for patient safety in recognition of the seriousness of this problem (Joint Commission, 2022). [9–10] This reiteration emphasizes how crucial it is to address alarm fatigue in healthcare settings in order to protect the wellbeing and safety of patients.

Critical care units are widely recognized as highly stressful work environments, attributed to several factors including demanding and complex job descriptions, escalating admissions, unpredictability schedule changes, unrealistic expectations from patients and their families, and the frequent encounters with moral and end-of-life dilemmas. Nurses within these units contend with extended work hours, time constrains, limited breaks, staffing shortage, and substandard working conditions [7, 8]. Research consistently associated work-related stress with diminished performance, reduced quality of life, and a host of health issues encompassing physical, psychological, and interpersonal problems [9, 10]. Given the overwhelming responsibilities and demands associated with providing care in today’s healthcare systems, especially in critical care units, nurses are frequently exposed to high levels of stress [17]. Additionally, studies have consistently shown that nurses working in critical care units experience significantly higher levels of stress compared to their counterparts in other nursing specialties [18, 19]. Consequently, prolonged exposure to such heightened stress levels poses a grave risk to patient safety and the overall care quality of care provided [20]. 

When multiple alarms in the critical care unit sound simultaneously, nurses often feel overburdened. A monitor that emits unnecessary sounds or false alert has been identified as a stress-inducing factors for healthcare providers [21]. According to previous studies, a significant and positive correlation has been observed between the socio-demographic characteristics of nurses, such as work experience, education level, age, gender, nurse-patient ratio and the occurrence of alarm fatigue [22–24]. Furthermore, there is abundant evidence indicating that psychological factors such as stress and anxiety among nurses, play a substantial role in contributing to alarm fatigue [25]. 

To our knowledge, no previous studies have addresses alarm fatigue in Palestine. Therefore, the aim of the current study is to examine alarm fatigue among nurses working in critical care units. Additionally, the study aims to assess the relationship between alarm fatigue and perceived stress. This knowledge can be used to create strategies aimed at mitigating the adverse consequences of alarm fatigue among critical care nurses. The adverse effects of alarm fatigue on the professional achievements of nurses and patients’ outcomes can be reduced by education, planning, and providing counseling to nurses regarding the factors that have the greatest impact on the development of alarm fatigue.

## **Methodology**

### Study design

Descriptive cross-sectional study was conducted. The target population (*N* = 251) included critical care nurses working in hospitals located in the northern and central regions of the West Bank, Palestine. Due to the constrains imposed by the October 7th war in Palestine, which rendered physical access to all hospitals challenging, data were gathered through online surveys. The research was carried out between November 2023 and January of 2024.

The researcher initiated contact with the nursing directors of each hospital. Subsequently, they obtained consent to distribute the survey to all nurses working in intensive care units (ICUs) via email and social media groups, with the assistance of the head of ICU nurses. An explanatory letter detailing the study goals and purpose was attached to the online survey to ensure data quality and accuracy during data collection process. Additionally, the researchers included a cell phone number in the explanatory letter to address any potential questions or concerns from the participants.

### Sample size

The required sample size was determined using an Excel calculation sheet developed by Abdallah (2024) [26], (available at: https://www.researchgate.net/publication/378550258_Sample_size_calculation_sheet) based on the formula by Daniel (1999) [27], which adjusts for finite population size as follows:

n* = $\frac{nN}{n+(N-1)}$, where $n=\frac{{\left({Z}_{\frac{\alpha }{2}}+{Z}_{\beta }\right)}^{2}\left[P\left(1-P\right)\right]}{{d}^{2}}$ for two-sided tests. *N* is the population size (= 251), *P* is the assumed population proportion (= 0.50), *d* is the margin of error (= 0.05), α is the significance level (= 0.05), and *β* is the probability of Type II error (= 0.20, i.e., statistical power = 80%). ${Z}_{\frac{\alpha }{2}}$and 𝑍_𝛽_ (1.64 and 1.28, respectively) are the standard normal values such that 𝑃(𝑍 ≥ ${Z}_{\frac{\alpha }{2}}$ ) = 𝛼/2 and 𝑃(𝑍 ≥ 𝑍_𝛽_) = 𝛽. The calculated sample size was 191. The actual sample included 187 ICU nurses who completed the questionnaire out of the 251 nurses targeted in the study (a response rate of 74.5%).

## Inclusion / exclusion criteria

All nurses working in the ICU units for at least one year in Palestinian hospitals in the central and northern regions of the West Bank were included in the study. Student nurses and nurses on leave such as maternity leave was excluded from the study.

### Tool of the study

1. Structured questionnaire consisting of demographic characteristics of the nurses including age, gender, marital status, experience, qualifications, type of hospital, hospital unit, nurse to patient ratio, type of work shift, and extra outside work.
2. Alarm Fatigue Questionnaire. It comprises 13 items. The tool was originally developed by Torabizadeh et al. (2017) [28] with approval obtained to use this scale for conducting the study. Answers to each item are given using the 5-Point Likert scale: “always”, “usually”, “sometimes”, “rarely”, and “never”. These are scored from 0 (never) to 4 (always) except items 1 and 9 which are scored reversely. The total score range of the questionnaire is between 8 (minimum) and 44 (maximum), with higher scores indicating a greater impact of alarm fatigue on nurse’s performance.
3. Perceived Stress Scale: is a classic stress assessment instrument. The tool, while originally developed in 1983, remains a popular choice to help understand how different situations affect feelings and perceived stress. The scale is publicly available and consists of 10 items which ask about feelings and thoughts during the last month [29].

The overall alarm fatigue scale (AFS) and perceived stress scale (PSS) scores were obtained as the sum of points of individual items and then converted into three categories using the sten (standard ten) scale (Canfield, 1951) [30]. As applied by Nagórska et al. (2021) [31], a final sten score of 1–4 is defined as low, 5–6 is defined as average, and 7–10 is defined as high.

After creating the questionnaire, it was presented to 5 nursing PhDs and experts in scientific research and the field of critical care nursing to assess its face and content validity and obtain comments and feedback. Cronbach’s Alpha was determined for the Alarm fatigue scale (13 items, Chronbach’s α = 0.60) and for the Perceived stress scale (10 items, Chronbach’s α = 0.73).

The questionnaire was translated into Arabic and reverse -translated to ensure accuracy. The validity of the tool was assessed by consulting five experts in the field: two university professors and three ICU nurses. They were asked to review and approve its validity, provide feedback on clarity, and assess whether the items reflected the main objectives of the study. Any necessary modifications were made based on their feedback.

### Pilot study

Twenty ICU nurses completed the questionnaire as a pre-test before the full data collection process. This allowed the nurses to identify any ambiguities in the questionnaire’s wording, estimate the response rate, determine the actual time required for completion, and assess the questionnaire’s suitability and validity. Completing the surveys takes approximately 15 to 20 min.

### Ethical approval

was obtained from Ministry of Health, Helsinki Committee in Palestine and from the Arab American University (Approval number: PHRC/HC/23). Participation in the study was entirely voluntary for nurses, and declining to participate would not result in any negative consequences or penalties. Furthermore, the study meticulously safeguarded the confidentiality of the participants by not disclosing any names or personal information, which were kept securely for research purposes only. Informed consent was obtained from all participants, and all procedures were conducted in compliance with the guidelines outlined in the Declaration of Helsinki.

### Data analysis

**Fig. 1:**
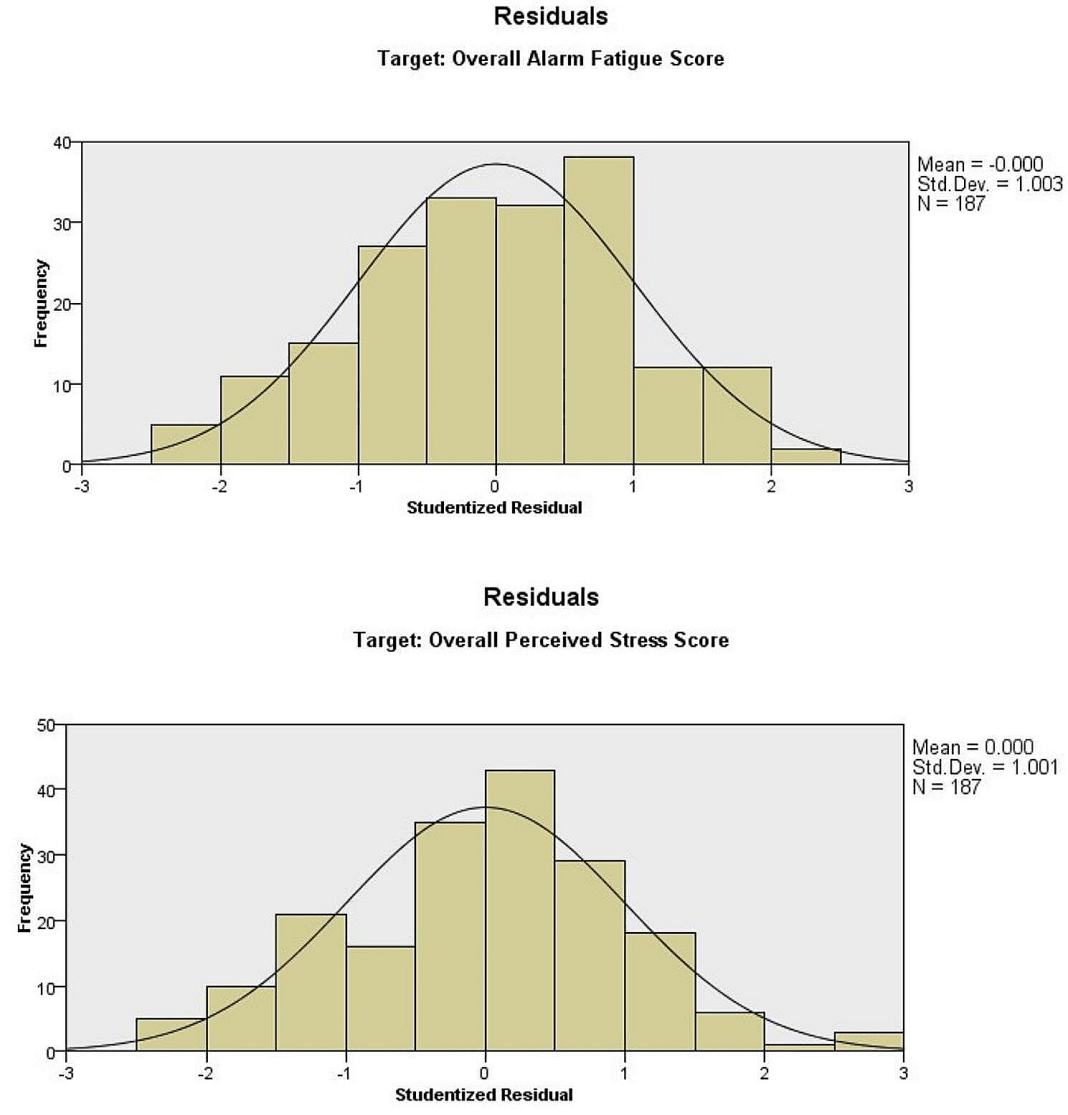
Histograms of studentized residuals from the final prediction models using Linear Automatic Modelling. The smooth line represents the normal distribution. The closer the frequencies of the residuals to the line, the closer the distribution of the residuals is to the normal distribution

The statistical analyses of data were conducted using the Statistical Package for Social Sciences (SPSS), version 21.0. Chronbach’s α was obtained as a measure of reliability. The distributions of the overall AFS and PSS scores were assessed graphically using Boxplots and Normal Probability Plots. Boxplots of the data showed symmetric distributions with no extreme values, and the Normal Probability Plots did not reveal any serious deviations from normality. Therefore, we adopted a parametric statistical approach for the analysis of data. Descriptive statistics (frequencies, percentages, means, and standard deviations) were used to summarize the data. Correlation analysis was performed to obtain Pearson correlation coefficients between the overall AFS and PSS scores, while Fisher’s Exact test was performed to test the association between AFS and PSS score categories. Independent samples t-test and one-way ANOVA were carried out to test differences in means of overall AFS and PSS scores among levels of categorical variables (e.g., socio-demographic, and other characteristics of participating nurses). The Linear Automatic Modeling regression function of SPSS was utilized to determine which factors are important predictors of alarm fatigue and perceived stress using the forward stepwise method based on the adjusted-R^2^ criterion for entry and removal of predictor variables. The factors assessed for prediction are summarized in Table 1. Assessment of the distribution of the studentized residuals from the final prediction models showed that the distributions are fairly close to the normal distribution (Fig. 1).

**Table 1:** Factors assessed for prediction of overall alarm fatigue scale (AFS) scores and perceived stress scale (PSS) scores using Linear Automatic Modeling regression

Prediction factors	Predicted variable	
	Overall AFS score	Overall PSS score
Age group	✔	✔
Gender	✔	✔
Marital status	✔	✔
Qualification	✔	✔
Type of hospital	✔	✔
Hospital unit	✔	✔
Experience	✔	✔
Nurse-to-patient ratio	✔	✔
Type of working shift	✔	✔
Extra outside work	✔	✔
AFS category		✔
PSS category	✔	

## Results 

### Alarm fatigue scale (AFS) 

Table 2 presents the means of individual item and overall AFS scores. The mean overall AFS score was 23.36 (SD = 5.57) out of 44. Among all participating nurses, 70 (37.4%) experience low alarm fatigue, 62 (33.2%) experience average alarm fatigue, and 55 (29.4%) experience high alarm fatigue.

The responses to individual items by the ICU nurses participating in the study indicated a greater impact of alarm fatigue on their performance when they were asked about item 5 “I pay more attention to the alarms in certain shifts” (3.58 out of 4), and item 4 “I believe much of the noise in the ward is from the alarms of the monitoring equipment ” (3.06 out of 4). On the other hand, the lowest impact of alarm fatigue on ICU nurses’ performance were for item 11 “When alarms go off repeatedly, I become in different to them” (0.80 out of 4), and item 2 “I turn off the alarms at the beginning of every shift” (0.82 out of 4).

Among all characteristics of participating nurses, only gender have a significant effect (*P* < 0.05) on the overall AFS score, with female nurses having a higher average score (24.60) than male nurses (22.65). All the other characteristics showed non-significant effects (*P* > 0.05), as shown in Table 3.

**Table 2:** Means of Individual item and overall alarm fatigue scale (AFS) scores

	Mini.	Max.	Mean	SD
Individual items				
1. “I regularly readjust the limits of alarms based on the clinical symptoms of patients”	0.00	3.00	0.90	0.91
2. “I turn off the alarms at the beginning of every shift”	0.00	4.00	0.82	1.17
3. “Generally, I hear a certain amount of noise in the ward”	1.00	4.00	2.92	0.85
4. “I believe much of the noise in the ward is from the alarms of the monitoring equipment”	1.00	4.00	3.06	0.81
5. “I pay more attention to the alarms in certain shifts”	1.00	4.00	3.58	0.76
6. “In some shifts the heavy workload in the ward prevents my quick response to alarms”	0.00	4.00	1.39	1.01
7. “When alarms go off repeatedly, I become in different to them”	0.00	4.00	0.80	1.07
8. “Alarm sounds make me nervous”	0.00	4.00	1.98	1.13
9. “I react differently to the low-volume (yellow) and high-volume (red) alarms of the ventilator”	0.00	4.00	0.95	0.97
10. “When I’m upset and nervous, I’m more responsive to alarm sounds”	0.00	4.00	1.81	1.17
11. “When alarms go off repeatedly and continuously, I lose my patience”	0.00	4.00	1.68	1.21
12. “Alarm sounds prevent me from focusing on my professional duties”	0.00	4.00	1.99	1.09
13. “At visiting hours, I pay less attention to the alarms of the equipment”	0.00	4.00	1.48	1.22
**AFS overall score**				
**AFS category**				
Low (*n* = 70, 37.4%)	10.0	20.0	17.78	2.85
Average (*n* = 62, 33.2%)	21.0	26.0	23.48	1.43
High (n **=** 55, 29.4%)	27.0	35.0	30.33	2.10
ALL (*N* = 187, 100%)	**10.0**	**35.0**	**23.36**	**5.57**

## Perceived stress scale (PSS)

The means of PSS scores for individual items and the overall scores for nurses working in the ICU units are presented in Table 3. The mean of the overall PSS scores was 16.99 out of 40. Among all participating nurses, 57 (30.5%) were categorized as having low perceived stress level, 73 (39.0%) were categorized as having an average perceived stress level, and 57(30.5%) were categorized as having a high perceived stress level.

The response of the ICU nurses to individual PSS items regarding their feelings and thoughts in the last month showed that the highest average scores were for item 3 “how often have you felt nervous and “stressed?” (2.09 out of 4), item 6 “how often have you found that you could not cope with all the things that you had to do?” (1.95out of 4), and item 1 “how often have you been upset because of something that happened unexpectedly” (1.88 out of 4). While the lowest averages for ICU nurse’s responses were for item 8 “how often have you felt that you were on top of things” (1.28 out of 4), for item 4 “how often have you felt confident about your ability to handle your personal problems?” (1.45 out of 4), and item 7 “how often have you been able to control irritations in your life?” (1.48 out of 4).

**Table 3:** Means of individual item and overall perceived stress scale (PSS) scores

	Min.	Max.	Mean	SD
Individual items				
1. “In the last month, how often have you been upset because of something that happened unexpectedly?”	0.00	4.00	1.88	0.85
2. “In the last month, how often have you felt that you were unable to control the important things in your life?”	0.00	4.00	1.79	0.91
3. “In the last month, how often have you felt nervous and “stressed”?	1.00	4.00	2.09	0.81
4. “In the last month, how often have you felt confident about your ability to handle your personal problems?”	0.00	4.00	1.45	0.85
5. “In the last month, how often have you felt that things were going your way”	0.00	3.00	1.62	0.71
6. “In the last month, how often have you found that you could not cope with all the things that you had to do?”	0.00	4.00	1.95	0.90
7. “In the last month, how often have you been able to control irritations in your life?	0.00	3.00	1.48	0.84
8. In the last month, how often have you felt that you were on top of things?”	0.00	3.00	1.28	0.82
9. “In the last month, how often have you been angered because of things that were outside of your control?”	0.00	4.00	1.81	0.94
10. “In the last month, how often have you felt difficulties were piling up so high that you could not overcome them?”	0.00	3.00	1.64	0.83
**PSS overall score**				
**PSS Category**				
Low (*n* = 57, 30.5%)	6.0	14.0	11.63	2.44
Average (*n* = 73, 39.0%)	15.0	19.0	17.19	1.40
High (*n* = 57, 30.5%)	20.0	26.0	22.09	1.76
All (*n* = 187, 100%)	**6.0**	**26.0**	**16.99**	**4.50**

The analysis of variance showed a statistically significant effect of the type of work shift on ICU nurses’ perceived stress (*P* < 0.05), where nurses working double shifts had the lowest average score (15.23), and nurses working regular morning shifts had the highest average score (17.92). Conversely, there were no statistically significant effects (*P* > 0.05) of age, marital status, experience, qualification, hospital unit, nurse-to- patient ratio, and extra work on ICU nurses’ alarm fatigue and perceived stress (see Table 4).

**Table 4:** Means of overall alarm fatigue scores (AFS) and perceived stress scores (PSS) by characteristics of participating nurses

Characteristic	N (%)	AFS overall score		PSS overall score	
		Mean (SD)	P value^1^	Mean (SD)	P value^1^
Gender			0.021		0.485
Males	119 (63.6)	22.65(5.58)		16.82(4.63)	
Females	68 (36.4)	24.60(5.38)		16.82(4.63)	
**Age group**			**0.680**		**0.844**
≤ 25 years	25 (13.4)	23.32(5.65)		17.04(4.12)	
26–30 years	98 (52.4)	23.48(5.48)		16.77(4.52)	
31–35 years	35 (18.7)	23.94(5.32)		17.57(4.78)	
> 35 years	29 (15.5)	22.28(6.24)		17.00(4.59)	
**Marital status**			**0.889**		**0.119**
Married	94(50.3)	23.41(5.68)		17.50(4.47)	
Single	93(49.7)	23.30(5.49)		16.47(4.49)	
**Experience**			**0.432**		**0.717**
< 5 years	76(40.6)	23.30(5.89)		16.67(4.46)	
5–10 years	68(36.4)	23.94(5.10)		17.15(4.46)	
> 10 years	43(23.0)	22.53(5.75)		17.30(4.70)	
**Qualification**			**0.391**		**0.496**
Diploma	22(11.8)	22.95(5.65)		17.36(3.09)	
Bachelor	135(72.2)	23.14(5.58)		16.76(4.74)	
Master or higher	30(16.0)	24.63(5.51)		17.77(4.29)	
**Hospital unit**			**0.275**		**0.213**
Emergency care unit	15(8.0)	24.53(6.02)		16.93(4.48)	
General Critical care unit	83(44.4)	22.76(5.42)		16.59(4.71)	
Medical Critical unit	45(24.1)	23.98(5.37)		17.71(4.12)	
Surgical Cardiac unit	18(9.6)	21.72(4.14)		15.39(4.85)	
Other	26(13.9)	24.65(6.77)		18.15(4.02)	
**Nurse-to-patient ratio**			**0.078**		**0.577**
1: 1	7(3.7)	19.71(7.18)		15.57(7.00)	
1: 2	81(43.3)	22.65(5.69)		16.59(4.67)	
1: 3	61(32.6)	24.48(5.05)		17.13(4.39)	
1: 4	13(7.0)	22.23(5.13)		18.15(4.54)	
1: ≥5	25(13.4)	24.52(5.69)		17.72(3.32)	
**Type of work shift**			**0.301**		**0.019**
Double shift	39(20.9)	22.26(4.42)		15.23(3.94)	
Irregular shift	114(61.0)	23.47(5.77)		17.33(4.61)	
Regular evening shift	9(4.8)	24.22(6.16)		17.67(4.15)	
Regular morning shift	25(13.4)	24.24(6.11)		17.92(4.49)	
**Extra outside work**					
No	120(64.2)	23.23(5.40)	**0.683**	17.00(4.46)	**0.965**
Yes	67(35.8)	23.58(5.91)		16.97(4.61)	

^1^ P values from independent samples t-test and One-way ANOVA

### Relationship of alarm fatigue scores and perceived stress scores

A significant positive Pearson correlation coefficient of 0.40 was found between AFS and PSS overall scores (*P* < 0.01). Fisher’s Exact test showed a highly significant association (*P* < 0.001) between the AFS and PSS score categories. A high frequency of nurses in the low AFS score category were in the low PSS score category (50.9%) and vice versa (41.4%); a high frequency of nurses in the average AFS score category were in the average PSS score category (49.3%) and vice versa (58.1%); and a high frequency of nurses in the high AFS category were in the high PSS category (61.4) and vice versa (63.6%) (refer to Table 5). Furthermore, the results from One-way ANOVA showed significant differences (*P* < 0.001) among AFS categories in mean PSS scores where nurses in the high AFS category had a significantly higher mean PSS score (19.45) than nurses in the low and average AFS categories (15.53 and 16.45, respectively). Similarly, significant differences (*P* < 0.001) were found among PSS categories in mean AFS scores where nurses in the high PSS category had significantly higher mean AFS score (26.79) than nurses in the low and average AFS categories (21.37 and 22.23, respectively).

**Table 5:** AFS categories by PSS categories cross-tabulation and mean total scores

		PSS category				
		Low	Average	High	Total	P value ^1^
**AFS Category**						
Low	n	29	28	13	70 (37.4%)	**< 0.001**
	% within AFS category	41.4%	40.0%	18.6%		
	% within PSS category	50.9%	38.4%	22.8%		
Average	n	17	36	9	62 (33.2%)	
	% within AFS category	27.4%	58.1%	14.5%		
	% within PSS category	29.8%	49.3%	15.8%		
High	n	11	9	35	55 (29.4%)	
	% within AFS category	20.0%	16.4%	63.6%		
	% within PSS category	19.3%	12.3%	61.4%		
Total	n (%)	57 (30.5%)	73 (39.0%)	57 (30.5%)	187 (100%)	
**PSS overall scores**						
**AFS category**		**Mean**	**SD**			P value ^2^
Low		15.53^b,3^	4.58			**< 0.001**
Average		16.45^b^	4.37			
High		19.45^a^	3.48			
**AFS overall scores**						
**PSS category**		**Mean**	**SD**			P value ^2^
Low		21.37 ^b, 3^	5.99			**< 0.001**
Average		22.23 ^b^	3.71			
High		26.79 ^a^	5.66			

^1^ P value based on Fisher’s Exact test of association between AFS and PSS categories

^2^ P values based on One-way ANOVA, test if differences exist among categories in mean AFS and PSS scores

^3^ Means in the same column with different superscripts are significantly different (*P* < 0.05) based on Fisher’s Protected LSD test for multiple comparisons

### Prediction model for the overall AFS (alarm fatigue scale) scores

**Fig. 2:**
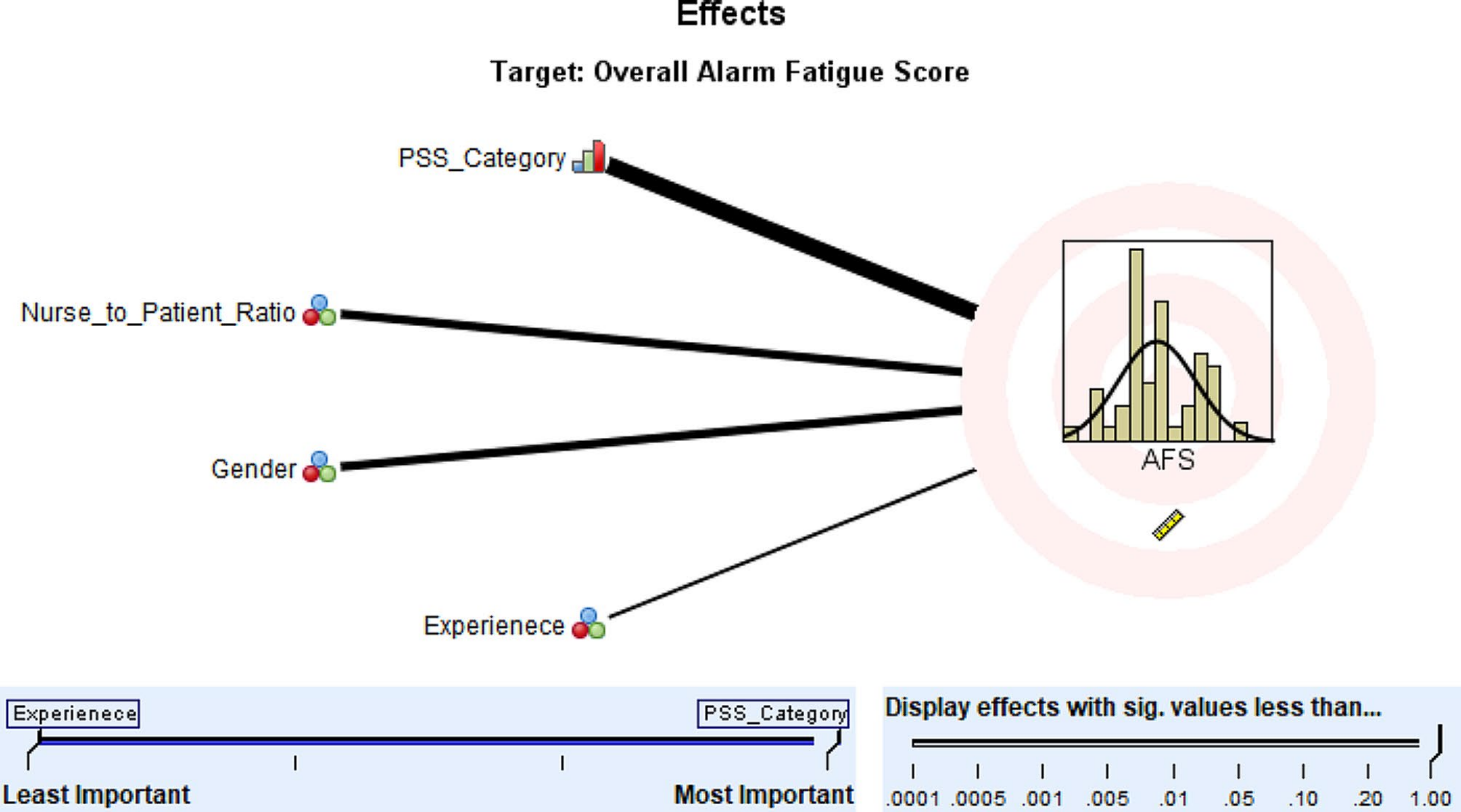
Important predictors of overall AFS (Alarm Fatigue Scale) scores of nurses working in the critical care units in Palestine

The variables retained for predicting the AFS overall score using the Linear Automatic Modeling procedure included (in order of importance): PSS score category (*P* < 0.001, importance = 0.689), nurse-to-patient ratio (*P* = 0.034, importance = 0.176), gender (*P* = 0.024, importance = 0.085), and experience (*P* = 0.216, importance = 0.051) with adjusted R^2^ = 0. 217 (Fig. 2; Table 6). Nurses in the low and average PSS score categories had -ve coefficients (− 5.533 and − 4.706, respectively) compared to those in the high PSS score category; nurses caring for fewer than 5 patients had–ve coefficients compared to those caring for 5 or more patients; female nurses had + ve coefficient (1.732) compared to male nurses; and nurses with less than 5 year experiences and those with 5–10 year experience have + ve coefficients compared to those with more than 10-year experience (see Table 6).

**Table 6:** Prediction model for the overall AFS (Alarm Fatigue Scale) scores

Model term	Coefficient	P value	Importance
Intercept	26.377	< 0.001	
PSS category		< 0.001	0.689
Low	-5.533***		
Average	-4.706***		
High	0 ^a^		
Nurse-to-patient ratio		0.034	0.176
1: 1	-3.715*		
1: 2	-1.799*		
1: 3	0.063*		
1: 4	-3.261*		
1: ≥5	0 ^a^		
Gender		0.024	0.085
Female	1.732**		
Male	0 ^a^		
Experience		0.216	0.051
< 5 years	0.910*		
5–10 years	1.731*		
> 10 years	0 ^a^		

^a^ These coefficients are set to 0 because they are redundant (the coefficients of the other levels are interpreted as differences from this coefficient)

*** *P* ≤ 0.01, ** *P* ≤ 0.05, * *P* > 0.05: tests the difference between this coefficient and the coefficient of the last category that is set to 0

### Prediction model for the overall PSS (perceived stress scale) scores

**Fig. 3:**
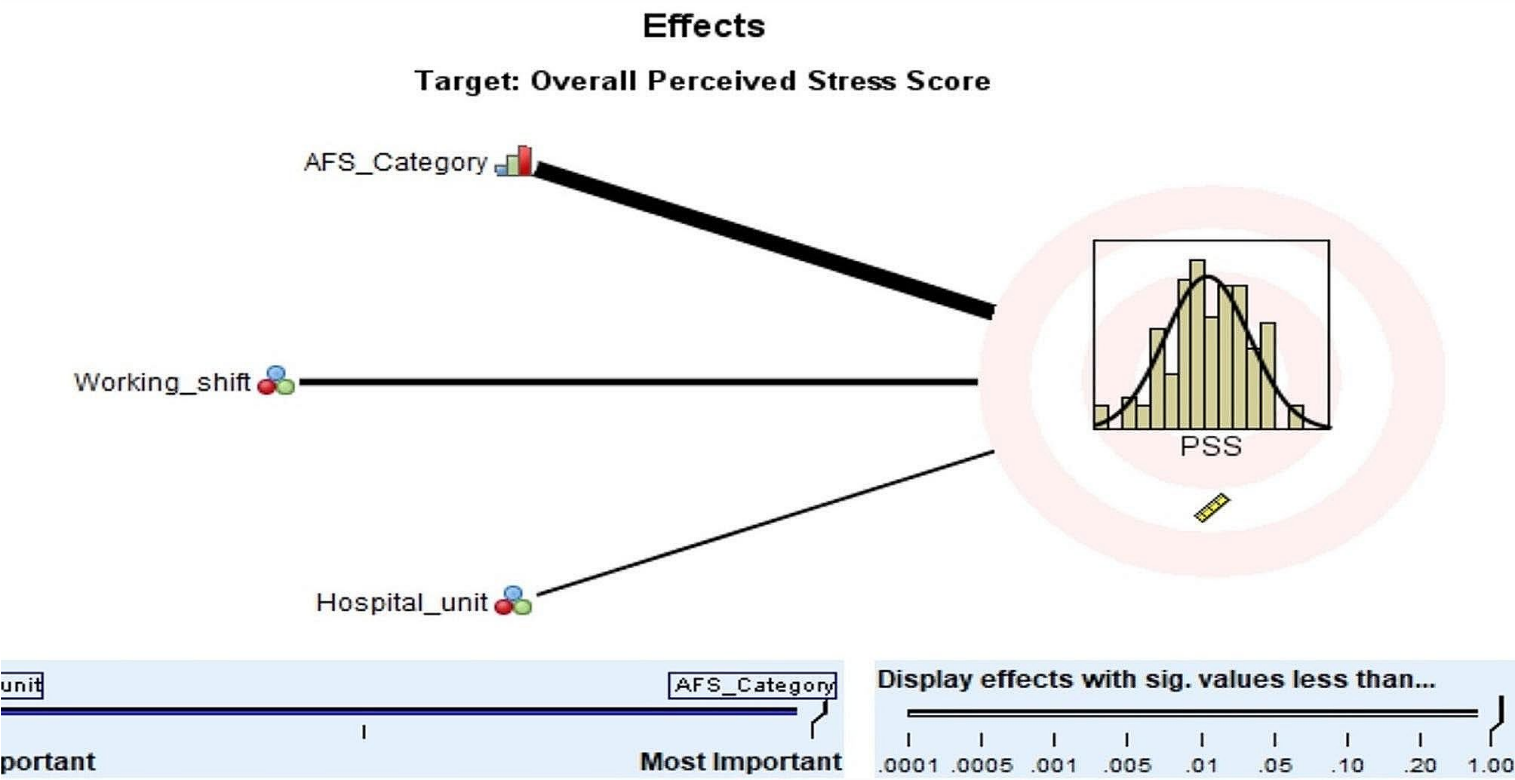
Important predictors of overall PSS (Perceived Stress Scale) scores of nurses working in the critical care units in Palestine

For predicting the PSS overall score, the prediction model included AFS score category (*P* < 0.001, importance = 0.674), type of working shift (*P* = 0.099, importance = 0.195), and hospital unit (*P* = 0.375, importance = 0.131), with adjusted R^2^ = 0.138 (Fig. 3; Table 7). Nurses in the low and average AFS score categories have -ve coefficients (− 3.582 and − 2.463, respectively) compared to those in the high AFS score category; nurses working double shifts and irregular shifts had–ve coefficients compared to those working regular morning shifts (-2.144 and − 0.288, respectively) while nurses working regular evening shifts had a slight positive coefficient (0.029); nurses working in the emergency care units, general critical care units, medical critical care units, and other types of hospital units had positive coefficients (0.619, 0.655, 1.566, and 2.114, respectively) compared to nurses working in the surgical cardiac units, (Table 7).

**Table 7:** Prediction model for the overall PSS (Perceived Stress Scale) scores

Model term	Coefficient	P value	Importance
Intercept	18.756	< 0.001	
AFS category		< 0.001	0.674
Low	-3.582***		
Average	-2.463***		
High	0 ^a^		
Working shift		0.034	0.195
Double shift	-2.144**		
Irregular shift	-0.288*		
Regular evening	0.029*		
Regular morning	0 ^a^		
Hospital Unit		0.375	0.131
Emergency Care unit	0.619*		
General Critical Care unit	0.655*		
Medical Critical Care unit	1.566*		
Other units	2.114*		
Surgical Cardiac	0 ^a^		

^a^ These coefficients are set to 0 because they are redundant (the coefficients of the other levels are interpreted as differences from this coefficient)

*** *P* ≤ 0.01, ** *P* ≤ 0.05, * *P* > 0.05: tests the difference between this coefficient and the coefficient of the last category that is set to 0

## Discussion

To the best of our knowledge, no previous study has been conducted to examine the impact of alarm fatigue on nurses’ performance, its associated factors, and its relationship with perceived stress in Palestine. Our study revealed that the mean total score of the Alarm Fatigue Scale was 23.36, indicating that critical care nurses in Palestine experience moderate levels of alarm fatigue. Critical care nurses are particularly susceptible to alarm fatigue due to the extensive time they devote for patient care and monitoring, necessitating continuous vigilance and prompt response to alarms generated by numerous medical devices [14]. Consequently, health care professionals may develop a desensitization to the frequent alarm occurrences. This desensitization could lead to crucial alarms being missed or responded to slowly, resulting in decrease concentration and physical exhaustion, thereby jeopardizing patient safety and wellbeing, potentially leading to adverse outcomes, including patient mortality. These findings are in line with studies conducted in Iran, Italy, and China [32–34]. Conversely, a study conducted in Ghana found that the majority of nurses experienced severe alarm fatigue [35]. 

Additionally, our findings revealed that the item with the highest average score was “I pay more attention to the alarms in certain shifts”. This tendency may be attributed to the nature of work in intensive care units, where alarm fatigue is prevalent, and behaviors during specific shifts may be influenced by factors such as nurse-patient ratio, patient acuity, and varying workload. For example, during morning shifts, the workload on nurses may increase, leading to heightened attention to alarms [36]. Conversely, in Korea the highest scoring item was “I hear a certain amount of noise in the ward” [38]. The items with the lowest scores were “I turn off the alarms at the beginning of every shift” and “I regularly readjust the limits of alarms based on the clinical symptoms of patients”. These findings sugest that ICU nurses, despite experiencing alarm fatigue, promptly respond to any alterations in patients’ conditions. These findings are consistent with previous studies [37, 38]. However, they contrast with a study conducted in Ireland, where the majority of nurses either disregarded the alarm or exhibited delayed responses when experiencing alarm fatigue [39]. 

Another important finding of this study was that the majority of respondents reported an average to high level of perceived stress. In addition to work and social factors, this can also be attributed to the prolonged conflict between Israel and Palestine, which increases the risk of anxiety and stress among Palestinians [40]. Furthermore, healthcare professionals in Palestine are more likely to experience stress and burnout when working in an extremely restricted healthcare system under Israeli military occupation [41, 42]. This finding aligns with a previous study conducted in India, which found that the majority of NICU nurse experienced a moderate level of stress [43]. In contrast to our findings, a previous study conducted in Qatar reported that nurses experienced a high level of perceived stress [44]. Given the demanding nature of the healthcare professionals, nurses are particularly susceptible to issues related to occupational stress [45]. 

The PSS Score category emerged as the most important predictor of the AFS overall score and vice versa (the AFS Score category emerged as the most important predictor of the PSS overall score). Nurses in the low and average PSS score categories are expected to have lower AFS scores than the high PSS category by an average of 5.53 and 4.71 points, respectively. Nurses in the low and average PSS score categories are expected to have lower AFS scores than the high PSS category by an average of 3.582 and 2.463 points, respectively This suggests that ICU noises may be stressful to healthcare professionals [46], but also suggests that stressed nurses may have aggravated alarm fatigue. Research has shown that loud noise in intensive care units (ICUs) can negatively impact health care professionals, leading to increased stress and decreased performance [47]. Alarms contribute to this noise and can be disruptive to nurses, hindering their ability to effectively carry out their tasks [48]. Therefore, it is essential to reduce noise-induced stress among critical care nurses to improve the working environment and patient care [49]. Furthermore, healthcare institutions should consider evaluating their alarm policies and procedures, potentially integrating artificial intelligence technologies for more efficient alarm management.

In intensive care units, the utilization of numerous medical devices contributes to the escalating noise level and increasingly loud alarms, which are reported psychological issues by nurses [38]. Health professionals exposed to this sensory overload for extended periods of time experience both cognitive and physical stress [50, 51]. 

Additionally, nurse- to-patient ratio and gender were identified as predictors of alarm fatigue, with female nurses being more susceptible experiencing it. This could be attributed to the heavier workload carried by female ICU nurses, not only due to stressful working environment but also compounded by personal responsibilities such as housework, childcare, and balancing work -life commitments [36, 52]. Our results align with a previous study, which also indicated a higher likelihood of alarm fatigue among female nurses [32]. However, this contrasts with the findings of Bourji H, et al. (2020), which found that male nurses experience alarm fatigue more frequently than female nurses [25]. 

The study’s results also revealed that nurses working regular morning shifts experienced high levels of perceived stress. This could be attributed to the heavy workload of morning shift nurses, which includes tasks such as patient admission and discharge, patient care responsibilities, and managing visitors. Occasionally, these demands may lead them to skip their breaks [53–56]. 

## Limitation

A Self-administered survey was considered a limitation in this study. Future research should consider employing observational checklist and qualitative research designs to address alarm fatigue more accurately among critical care nurses. Furthermore, certain factors were not addressed, such as the consequences of alarm fatigue on quality of patient care, resilience, and burnout. These aspects warrant further investigation in future studies.

## Implications for clinical practice

To mitigate alarm fatigue among critical care nurses, managers should implement comprehensive alarm management protocols, including regular adjustment of alarm settings and the integration of artificial intelligence technologies for efficient alarm management. Continuous training and education programs on alarm management best practices are also essential. Addressing nurses’ stress levels is imperative to ensure patient safety and improve the quality of care. Identifying sources of stress and implementing effective stress reduction intervention, such as mindfulness-based techniques, is crucial. Further studies are warranted to explore these interventions. Additionally, it is essential to assess the complex relationship between alarm fatigue, stress and patient outcomes. This will facilitate the development of evidence-based practices and policies that support critical care nurses and enhance patient care outcomes.

## Conclusion

Alarm fatigue can compromise the timely intervention necessary to prevent adverse outcomes by causing delayed responses or missed critical alarm, thereby posing a major risk for patient safety. The study indicated that the highest score was related to nurses’ attention to alarm varying according to shift, a factor not addressed in the current study. Therefore, future research should examine the impact of different shifts on alarm fatigue and patient outcomes. Additionally, it is imperative that healthcare providers address stress, as it has been found to be an important predictor of alarm fatigue. This will aid in mitigating alarm fatigue and fostering a supportive work environment conducive to providing optimal patient care. Furthermore, future research studies are needed to assess the effectiveness of educational training in managing alarm fatigue and reducing stress levels among critical care nurses.

## Data Availability

The data utilized to support the results of the research are accessible to the corresponding author upon request.

## Abbreviations

*ICU*: Intensive Care Unit
*AFS*: Alarm Fatigue Scale
*PSS*: Perceived Stress Scale
